# The wound healing effect of botanicals and pure natural substances used in in vivo models

**DOI:** 10.1007/s10787-023-01157-5

**Published:** 2023-02-22

**Authors:** S. A. El-Sherbeni, W. A. Negm

**Affiliations:** Department of Pharmacognosy, Faculty of Pharmacy, Tanta University, Tanta, 31527 Egypt

**Keywords:** Animal models, Burns, Inflammation, Natural products, Wound healing, Wound dressings

## Abstract

Repairing the wound is a multistep process that includes the spatial and temporal synchronization of a different range of cell types to increase the speed of wound contraction, the proliferation of epithelial cells, and collagen formation. The need for proper management of acute wounds to be cured and not turned into chronic wounds is a significant clinical challenge. The traditional practice of medicinal plants in many regions of the world has been used in wound healing since ancient times. Recent scientific research introduced evidence of the efficacy of medicinal plants, their phyto-components, and the mechanisms underlying their wound-repairing activity. This review aims to briefly highlight the wound-curing effect of different plant extracts and purely natural substances in excision, incision, and burn experimental animal models with or without infection of mice, rats (diabetic and nondiabetic), and rabbits in the last 5 years. The in vivo studies represented reliable evidence of how powerful natural products are in healing wounds properly. They have good scavenging activity against Reactive oxygen species (ROS) and anti-inflammatory and antimicrobial effects that help in the process of wound healing. It is evident that incorporating bioactive natural products into wound dressings of bio- or synthetic polymers in nanofiber, hydrogel, film, scaffold, and sponge forms showed promising results in different phases of the wound-curing process of haemostasis, inflammation, growth, re-epithelialization, and remodelling.

## Introduction

The human body includes different organs. One of them is the skin which occupies a large body area. It represents the outermost defensive covering of the body and an immunological barrier that regularly faces different external factors. It fortifies against mechanical pressure, microbial contagion, and septicity and maintains normal body temperature. It is responsible for the sensation of touch, heat, and cold (Richmond and Harris 2014; Kwiecien et al. 2019; Kumar P and Kothari 2021).

The antimicrobial protective role of different skin layers was evidenced through different previous studies. An external layer displays the composition of human skin outside the epidermis called microbiota, epidermis, dermis, adipose tissue, glands (sweat and sebaceous), and hair follicles (Kwiecien et al. 2019).

Epidermis is composed of keratinocytes, melanocytes, Langerhans’ cells, and Merkel cells. Keratinocytes are a significant type of cells that has a role in vitamin D formation and produce keratin and lipids to form a water barrier. Keratinocytes could act against chemical and biochemical toxins by creating pro-inflammatory cytokines, e.g., interleukins: IL-1α, IL-1β, IL-3, and IL-6, interferons-alpha and beta, transforming growth factors, tumour necrosis factors, and others (Blume-Peytavi et al. 2016). Melanocytes are responsible for skin pigmentation. The first line of protectors of the skin is represented by Langerhans cells. They transport antigens in the skin to the lymph node. The membranes of Merkel cells interact with free nerve endings in the skin, so they have a sensory function. The dermis layer includes the sweat glands, blood vessels, muscles, and sensory neurons (Yousef et al. 2017). Symbiotic microorganisms of bacteria and fungi are recognized as skin colonies with harmless and vital effects in protecting the skin. They are inside hair follicles, sweat and sebaceous glands to protect the skin against invasive and microbial pathogens. Among them, species of Staphylococcus, Malassezia, Demodex folliculorum, and Demodex brevis were the most important (Grice and Segre 2011; Ibrahim et al. 2020).

Wounds have happened due to the loss of histological composition of the skin tissue due to internal or external factors or sequential loss of function in any layer of the skin, which leads to tissue disturbance (Herman and Bordoni 2020). The existence of wounds permits the entrance of different microbial agents as bacteria and viruses or any foreign elements, into the body. Inflammation of skin wounds is happened because of local microbial infections. Also, a generalized systemic infection (septicemia) could be found, a life-threatening condition (Percival 2002). Consequently, more research should be done to find out simple and effective ways of taking care of skin wounds to heal properly. The main goals are to stop bleeding, get rid of microbial infection of wounds, and help wounds to heal effectively without any complications or deformities (Sarabahi et al. 2012; Jones 2015).

Once any damage has occurred to the skin tissue, multiple cellular and extracellular pathways act in a harmonized way, and their functions must be performed in the appropriate order at a suitable time to achieve repair, growth, and tissue regeneration (Richmond and Harris 2014).

Bleeding due to damaged blood vessels must be stopped, which is considered the initial reaction in the process of wound repair, besides platelet stimulation to compose a fibrin clot. Immediately after that, the disturbed tissues discharge growth factors and pro-inflammatory cytokines. Upon controlling the bleeding, many inflammatory cells such as monocytes, macrophages, and neutrophils are gathered at the wound site to provoke the inflammatory response (inflammatory phase). Moreover, the different self and exogenous antigens trigger the immune system to fight against them (Rodrigues et al. 2019; Alotaibi et al. 2021).

**Fig. 1:**
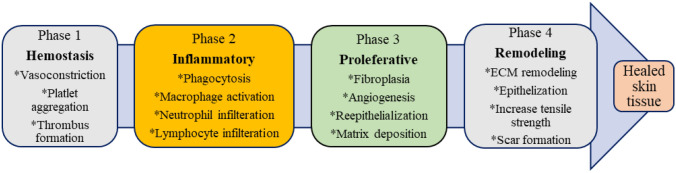
Different phases of skin wound healing

**Fig. 2:**
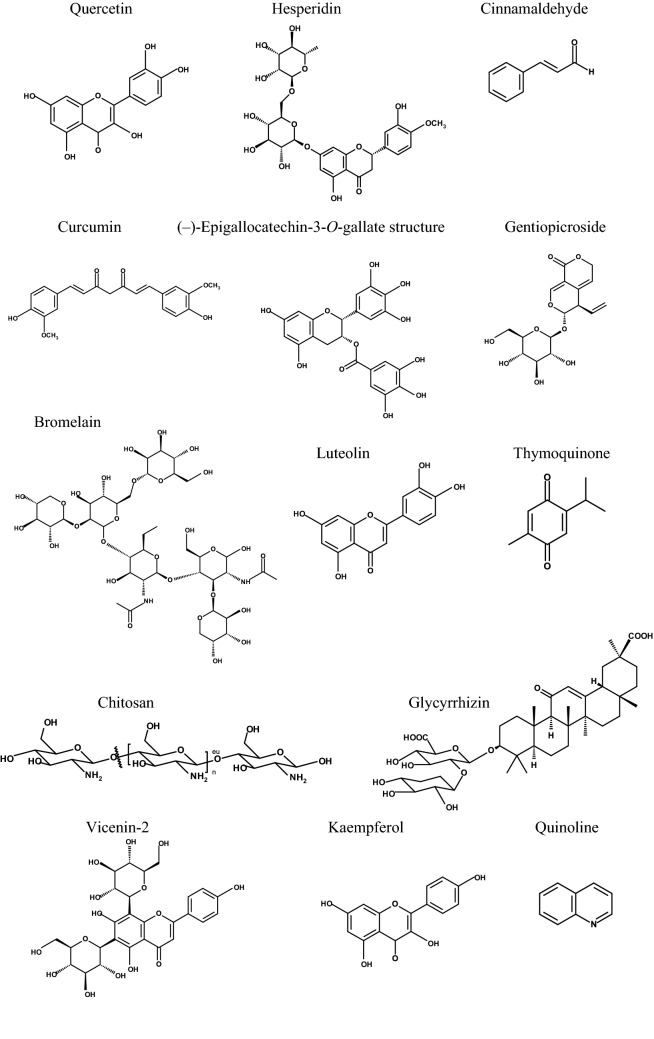
Structures of natural pure substances were investigated using wound healing in vivo models (animal models)

Angiogenesis is the following phase, which is parallel to the inflammation phase. The formation of a new blood vessel characterizes this phase. It is then followed by the growth and proliferative phases, which are predominate by fibroblast relocation and propagation, production of the matrix proteins, keratinocyte proliferation, differentiation, and restoration of hair follicles, etc. lastly, the wound healing process is finished with the remodelling of the extracellular matrix (ECM), besides the reordering of granulation tissue to scar tissue. Collagen synthesis and cross-linking afford stability to the healing tissue (Rodrigues et al. 2019). Figure 1 demonstrates the different phases of wound healing, while Table 1 summarizes herbal extracts studied using in vivo wound healing models. Structures of purely natural substances that were investigated using wound healing in vivo models showed in Fig. 2 and Table 2.

**Table 1:** Botanical extracts investigated by wound healing in vivo models (animal models)

No.	Plant name and part used	Family	Wound model and treatment	Animal	Outcome	References
1	*Aloe megalacantha* Baker (Leaf latex)	*Xanthorrhoeaceae*	Leaf latex was loaded to an ointment base (5% and 10% w/w)	Using for incision Swiss albino mice and excision Sprague Dawley rats' models	Both wound models showed a significant increase in the speed of wound contraction, epithelial cell proliferation, and increased tensile strength	Gebremeskel et al. 2018
2	*Phyllanthus muellerianus* (water extract of aerial part and pure compound geraniin)	*Euphorbiaceae*	Aqueous creams of the plant (0.25, 0.5, and 1% w/w) and geraniin (0.1, 0.2, and 0.4% w/w)	Male Sprague–Dawley rats were used with induced excision and incision wounds	A Remarkable elevation in fibroblasts, cross-linking, and collagen content in *P. muellerianus* and geraniin-treated wound tissues were shown. Also, notable levels of TGF-β_1_ were recorded	Boakye et al. 2018
3	The biofunctionalized silver nanoparticle was produced from cloves extract	*Myrtaceae*	AgNP was loaded into a cream with concentrations of 3% and 5%	Excision and incision male and female albino rats wound models	The wound-repairing impact was notable in animals treated with 5% silver nanoparticles. Collagen	Parveen et al. 2018
4	*Euphorbia characias* subsp. Wulfenii	*Euphorbiaceae*	1% ointment of methanol *n*-hexane, and ethyl acetate extracts *E. characias* subsp.	It was investigated in the linear incision and circular excision wounds in male Sprague Dawley rats	*E. characias* subsp. wulfenii displayed significant wound-curing activity	Özbilgin et al. 2018
5	*Lafoensia pacari* A. St.-Hil	*Lythraceae*	The hydroethanolic leaves extract was tested at 10, 30, or 100 mg/g of gel	Excision and incision-rat (Rattus norvegicus, Wistar strain model)	Increased rates of wound contraction, moderate re-epithelialization, neovascularization, proliferation, and acceleration of the remodeling phase	Pereira et al. 2018
6	*Alkanna strigose*	*Boraginaceae*	(Hexane extract of roots) Rattus norvgecis model	Excision and incision Albino Wistar rats	The beneficial effect of *A. strigosa* extract was confirmed	Aburjai et al. 2019
7	*Vitis labrusca* (Hydroalcoholic extract of leaves)	*Vitaceae*	Oral administration of the extract at 100, 200, and 300 mg/kg	Excision wounds male Wistar rats	Histological evidence showed that the extract could be a potential oral medicine for healing purposes	Santos et al. 2021
8	*Coccinia grandis* (Polyphenol- rich fraction was obtained from the methanol extract of leaves)	*Cucurbitaceae*	The hydrogel of *Coccinia grandis* (1.5 mg/ g) was tested	Excision wounds in male albino rats which was infected by *B. cereus*	Polyphenolic compounds of *Coccinia grandis* could be utilized as a natural wound-repairing drug	Al-Madhagy et al. 2019
9	*Jacaranda decurrens* (Hydroalcoholic extract of leaves.)	*Bignoniaceae*	15 mg/g of extract in the ointment base was tested	Mice-excision wound model	The extract increases rate of wound curing by modulating the action of pro-inflammatory cytokines	Serra et al. 2020
10	*Phlomis russeliana (n*-hexane fraction of methanol extract of the aerial part.)	*Lamiaceae*	5% of the extract in carbopol- hydroxypropyl gel	excisional wound model in Swiss mice (Mus musculus)	*Phlomis russeliana* has a wound-healing effect following the ethnobotanical application	Okur et al. 2020
11	*Plumeria obtusa* (Ethanolic extract of leaves)	*Apocynaceae*	2.5, 5, and 10% spray of the plant extract	Excision wound Swiss albino Wistar rats model	The formula with 10% *P*. *rutic* extract spray showed the best wound healing effect	Bihani and Mhaske 2020
12	*Boerhavia diffusa* (Methanol and chloroform extracts of the leaves)	*Nyctaginaceae*	Ointment (10% w/v) of methanol or chloroform extracts	Excision wound assays in a Albino Wistar rat model	The methanol extract of *B. diffusa* have a significant wound-healing effect	Juneja et al. 2020
13	*Ephedra ciliata* (methanol extract and quercetin)	*Ephedraceae*	5, 10, 20% cream of *Ephedra ciliata* methanol extract and 20% quercetin	Albino male and female rat model with excision and burn wounds was used	The extract rich with quercetin (methanol extract) of *Ephedra ciliata* promoted natural wound healing. The healing effects of the 20% methanol extract were comparable to the 20% quercetin	(Yaseen et al. 2020)
14	*Moringa oliefera* (Hexane extract of seeds	*Moringaceae*	5% and 10% hydrogel of *n*-hexane extracts of Moringa oleifera seeds	Excision and incision Male Swiss albino mice wound healing model	The hydrogel containing *n*-hexane extract of *Moringa oleifera* seeds could act as a wound-healing agent	Ali et al. 2021
15	*Moringa oleifera* leaves	*Moringaceae*	Moringa leaves extract gel	Incision wound male Wister rat (*Rattus norvegicus*) model	*Moringa oleifera* leaves extract gel exerted wound healing effect by speeding epithelialization	Ayu et al. 2020
16	*Curatella americana* Linn. (Hydroethanolic extract of leaves) (HECA)	*Dilleniaceae*	lyophilized extract of *C. americana* 0.5 and 1% loaded to a gel	Excision Adult Swiss albino mice wound model	treatment with 1% of the extract displayed the highest wound-repairing effect	Fujishima et al. 2020
17	*Nigella sativa* oil	*Ranunculaceae*	Mats of polyurethane electrospun nanofibrous loaded with *Nigella sativa* oil were tested as wound healing dressing	The full-thickness excisional wound in female Sprague Dawley rats	The mat of *N. sativa*-loaded Polyurethane nanofibrous, significantly provoked the wound-healing process	Aras et al. 2021 Nordin et al. 2019
18	*Dodonaea viscosa* (Leaves methanol and chloroform extracts)	*Sapindaceae*	10% w/w herbal. Ointment of the extracts	Incision and excision Sprague Dawley rats wound models	Methanolic extract significantly accelerated the epithelization of the excision wound. The extracts exerted a notable elevation in the tensile strength regarding the incision model	Nayeem et al. 2021
19	*Roylea elegans* (Aqueous leaves extract)	*Lamiaceae*	The cream contained 5 or 10% of the aqueous extract of leaves	Burn Wistar albino rats model	*Roylea elegans* caused wound-healing acceleration	Upadhyay et al. 2021
20	*Cupressus macrocarpa* (Diethyl ether extract of leaves) (DEEL)	*Cupressaceae*	DEEL in 20% DMSO in normal saline was applied to wounded and infected rats by methicillin-resistant *Staphylococcus aureus* clinical isolates	Full-thickness excision wounds male albino rats	DEEL showed epidermis regeneration, granulation tissue maturation, and a decrease in inflammatory cell infiltration	Attallah NGM et al. 2021
21	*Zehneria scabra* (80% Methanol Leaf Extract)	*Cucurbitaceae*	5% and 10% (w/w) of 80% methanol extract in an ointment base	Incision and excision wounds in adult albino mice	*Z. scabra* exerted significant wound-repairing activity	Tekleyes et al. 2021
22	*Bersama abyssinica* (Hydro-methanol, chloroform, hexane, and water fractions of leaves)	*Francoaceae*	5% and 10% w/w ointment of the hydro-methanolic extract was investigated	Excision, incision, and burn wounds in adult Swiss albino mice	Both 5% and 10% w/w of hydro-methanolic extract and solvent fractions of the plant have wound-curing effects	Taddese et al. 2021
23	*Semecarpus anacardium L., Argemone mexicana L., Cocculus hirsutus L.,* and *Woodfordia fruticose K*	*Anacardiaceae Papaveraceaei Menispermaceae Lythraceae*	The polyherbalBhallatakadi Ghrita (BG) formulation is composed of this mixture	Incision and excision Wistar rats model	Quercetin, gallic acid, and fatty acids increased the healing rate by the *ghrita* formulation	Wayal and Gurav 2021
24	*Elaeis guineensis Jacq* (Leaves)	*Arecaceae*	Leave extracts	Sprague Dawley rats were used for making excision wounds with microbial infection	*E. guineensis* promote the healing of wounds even though they were infected, confirming its traditional use in wound curing	Rajoo et al. 2021
25	*Vernonia auriculifera Hiern* (methanol extract of leaves and its fractions	*Asteraceae*	Ointment preparations of 5% and 10% w/w of methanol and other fractions	Excision, incision, and burn wound models in Swiss albino mice and female Wistar rats	The plant’s different extracts (methanol, aqueous, and ethyl acetate) showed	Lambebo et al. 2021
26	*Brucea antidysentrica Rhamnus prinoides Dodonaea angustifolia*	*Simaroubaceae*	*Brucea antidysentrica (*extract of roots bark*), Rhamnus prinoides (*leaves*),* and *Dodonaea angustifolia (*80% methanol extract*)*	Types of induced wounds in Swiss albino mice were excision and incision wounds	The traditional use of these plants in repairing wounds was confirmed. This plants increase wound contraction rate and tensile strength and decrease the time needed for efficient epithelialization	Tessema and Molla 2021
27	*Jatropha Neopauciflora* Pax Latex	*Euphorbiaceae*	latex (50%, 75%, and 100%)	Incisions were made in normal and diabetic male mice (Mus musculus) mice	*neopauciflora* could be beneficial for wound management in *diabetes mellitus* and speeds up and stimulates the wound-healing process	Hernandez-Hernandez et al. 2021
28	*Sanguisorba officinalis* Roots (the isolated Rhoifolin-Rich Fraction RRF)	*Rosoideae*	2% carbopol and hydroxypropyl cellulose gel of RRF	Full-thickness excision wound white albino rat model	RRF enhanced re-epithelization, angiogenesis, and shoed anti-bacterial, immunomodulatory, and anti-inflammatory activities	Negm et al. 2022
29	*Platycodon grandifloras* (Water extract of the dried tuberous roots)	*Campanulaceae*	The concentrated water extract was mixed with medical vaseline to make an ointment. 10% *P. grandiflorus* mixed emulsifiable paste was tested	Scald model males specific-pathogen-free (SPF) Sprague–Dawley rat	*P. grandiflorus* showed a significant healing effect on cutaneous scald lesions. A well-repaired epidermis was observed in rats treated with *P. grandifloras*	Wang et al. 2022
30	*Pistacia vera* (Italian and Algerian oleoresins)	*Anacardiaceae*	Oleoresins mixed with vaseline (5% w/w)	Circular wound excision ew Zealand albino rabbits model	Both oleoresins had very high wound-healing activity agents	Boudjelal et al. 2022
31	*Moringa oleifera* (Hydroethanolic extract of seeds)	*Moringaceae*	5% and 10% of the extract of *Moringa oleifera* seeds is added to the hydrogel	Excision and incision wound Male Swiss albino mice models	Hydr-ethanolic extract of *M. oleifera* could be utilized in wound management as an alternative plan	Ali et al. 2022
32	*Calendula officinalis L.* (Flower extract)	*Asteraceae*	The wound dressing of collagen film containing flower extract	Excision wound male Sprague–Dawley rat model	The tested dressing for wound repair contained the calendula extract. It was loaded with collagen film and showed safe, stable, and effective effects	Rathod et al. 2022
33	*Curcuma longa* (Aqueous, 70% methanolic, and ethanolic extracts)	*Zingiberaceae*	Different extracts of *C. longa* encapsulated in Ethosome were tested to heal wounds.(0.25, 0.5, and 1 g/cm2)	Full-thickness skin wounds in adult Wistar rats	Encapsulation of *C. longa* led to a better shape of wound, and maturation of granulation tissue, with an accelerated rate of healing, compared to crude extract	Kumar S et al. 2022
34	*Globularia arabica* (Leaf methanol extract)	*Plantaginaceae*	The study used variable concentrations of *G*. *arabica* extract (1%, 5%, and 10%) in ointment base	Excision diabetic and nondiabetic male Wistar rat model	*G. arabica* could be useful in healing wounds by provoking collagen and hydroxyproline formation when added externally on the wounded skin	Alsarayreh et al. 2022
35	*Premna integrifolia* (Standardized extract)	*Lamiaceae*	5% (w/w) ointment of the standardized extract	Excision wound model in male and female Wistar albino rats	*Premna integrifolia* had a wound-healing impact and could contribute to curing the wounds as a source of bioactive constituents with wound-healing characteristics	Alsareii et al. 2022
36	*Zizyphus mauritiana*	*Rhamnaceae*	(Fruit extract)	Full-thickness excisional wounds in adult male New Zealand Dutch strain albino rabbits	ZFE might act as a potential alternative drug to speed wound repair due to its antioxidant and anti-inflammatory effects	Shady et al. 2022
37	*Parkia clappertoniana Keay* (Fruit husk extract)	*Fabaceae*	Ointment of fruit extract (0.3, 1, and 3%)	Excision wound model in male Sprague–Dawley rats	*P.clappertoniana* exerted wound-healing and antimicrobial effects	Kuma et al. 2022

**Table 2:** Natural pure substances investigated using wound healing in vivo models (animal models)

No.	Natural products derived substances	Wound model and treatment	Animal	Outcome	References
1	Quercetin	This combination is prepared by taking 15% carbopol and varying the gelatin ratio	Excision albino rat wound model.Multiple phases hydrogel system combined with quercetin loaded to liposomes	The rate of wound repair is raised, with a prominent decrease in wound closure time compared to the drug's dosage form	Jangde et al. 2018
2	Quercetin	Two ischemia–reperfusion (I/R) cycles were utilized in each animal to induce ulcer formation. Topical treatment was performed with 1 μmol/L quercetin in DMSO	The animal pressure ulcer mice model was established with two cycles	The treatment by quercetin caused a significant acceleration of wound closure, a reduction in immune cell infiltration, and pro-inflammatory cytokines formation	Yin et al. 2018
3	Quercetin	Different treatments of quercetin (0.3%) and quercetin-loaded chitosan nanoparticles (0.03%, 0.1%, 0.3%) in pluronic F-127 gel (20% w/v)	Excision male Wistar rat wound model	Quercetin nanoparticles at 0.03% showed significant hastening wound healing by affecting cytokines and growth factors in inflammatory and proliferative phases	Choudhary et al. 2020
4	Quercetin	Quercetin of 0.03, 0.1, and 0.3% in DMSO was tested	Excision wound adult male Wistar rat model	Modulation of growth factors, antioxidant parameters, and different cells of the wound healing process was confirmed by 0.3% quercetin	Kant et al. 2020a, b
5	Quercetin	quercetin at 10, 20, and 40 mg/mL concentrations in 10% DMSO	Diabetic Sprague Dawley rats, excision wounds,	Conversion from M1 to M2 phenotype by modulation of macrophage polarization led to inhibition of inflammation process by quercetin	Fu et al. 2020
6	Quercetin	Quercetin as well as the photo-stimulatory impact of low energy 632.8 nm laser irradiation, were tested. Quercetin was taken by oral gavage at 25 mg/kg b. w. in 5 ml of 1% carboxymethylcellulose (CMC) with or without low-level laser treatment	The wound type was an excisional wound used in nondiabetic and diabetic male albino rats	The quercetin combined with low-level laser treatment improves the wound-curing process more than the utilization of only one of them	Ahmed et al. 2018
7	Quercetin	Three different solvents were used to contain 0.3% of quercetin: corn oil, 10% DMSO, and ointment base	Excisional wounded adult male Wistar rats	The most efficient wound healing impact with an accelerated healing rate	Kant, Kumar, et al. 2020
8	Quercetin incorporated in a new scaffold:Polyethylene glycol (PEG)ylated graphene oxide/quercetin (GO-PEG/Que) and artificial acellular dermal matrix/quercetin (ADM-GO-PEG/Que)	1-Polyethylene glycol (PEG)ylated graphene oxide (GO-PEG) /quercetin. 2-Artificial acellular dermal matrix -GO-PEG/quercetin (0.1 mg/mL)	Excision wound model in diabetic male albino mice	Helps in collagen deposition and angiogenesis. ADM-GO-PEG/Que represents a new material for tissue engineering scaffold	Chu et al. 2018
9	Quercetin	Secondary intention wound healing model in Wistar rats. 0.2 ml of gel contained 5% quercetin, 5% benzocaine, and glycerin	Palatal wounds of 5 mm diameter in Wistar rats	A reduction in inflammatory cells and an elevation in fibroblast cells were observed	Taskan et al. 2019
10	Quercetin (QCN)- and oxygen-carrying 1-bromoperfluorooctane (PFOB)-loaded nano emulsions (QCN-NE and OXY-PFOBNE)	The hydrogel is containing LMWP-GFs/QCN-NE/OXY-PFOB-NE. Low-molecular-weight protamine (LMWP) /skin-permeable growth factors (GFs)	The type of wound was excisional in diabetic C57BL/6 mice (females)	The hydrogel elevated the wound repairing rate in the diabetic mice and downregulated wound size relative to the vehicle and LMWP-GFs. Nano-emulsion was produced to ameliorate the external delivery of quercetin and oxygen	Jee et al. 2019
11	Quercetin and ciprofloxacin	PCL-bases nanofiber loaded with ciprofloxacin hydrochloride and quercetin	A full-thickness excisional wound in male Wistar rats	The topical delivery of ciprofloxacin hydrochloride and quercetin functionalized nanofiber. Both drugs could act as a bioactive wound dressing substance	Ajmal et al. 2019a, b
12	Quercitrin and myricitrin were isolated from *Pistacia lentiscus* leaves	*Pistacia lentiscus* leaves methanol extract 5, 20 mg/mL. Quercitrin and myricitrin 1 mg/mL	Excisional wounds in male Wistar rats	PDL, quercitrin, and myricitrin efficiently impact the healing of skin wounds	Elloumi et al. 2022
13	Hesperidin	Alginate/chitosan containing different concentrations of hesperidin	Full-thickness excision in male Wistar rats	Hesperidin loaded to alginate/chitosan hydrogels can be utilized to treat skin wounds in humans	Bagher et al. 2020
14	Quercetin in nanofiber scaffold	Four treatments were tested:1-gauze, 2-Poly ε-caprolactone-gelatin, 3-Poly ε-caprolactone-gelatin-ciprofloxacin hydrochloride, and 4-Poly ε-caprolactone-gelatin-ciprofloxacin hydrochloride-quercetin nanofibers	Excision wounds in male Wistar rats	A new scaffold showed full repair of wounds, and it could be used as a dressing material for healing wounds	Ajmal et al. 2019a, b
15	Quercetin	20% quercetin	Excision wound albino rat model	Animal treated with quercetin and heparin sodium exhibited significant healing effects in comparison to the control group	Yaseen et al. 2020
16	Quercetin	Quercetin was loaded to polycaprolactone/gelatin electrospun nanofiber	Excision wound male Wistar rat model	Quercetin nanofibers treated wounds exhibited a significant wound contraction with upregulation of angiogenesis and collagen formation. These nanofibers provided good integrity and hydrophilicity for wound dressing applications	Karuppannan et al. 2022
17	A homogeneous polysaccharide (ZWP) from* Curcuma zedoaria*	Chitosan/silk hydrogel sponge loaded with platelet-rich plasma exosomes (PRP-Exos), ZWP, or PRP-Exos/ZWP	Excision wounds in diabetic emale Sprague Dawley rats	Wound contraction was recognized in the separate or combined treatments, as represented by a reduction in ulcer and an elevation in the thickness of epidermis. PRP-Exos/ZWP combined treatment gave better results in wound closure	Xu et al. 2018
18	Curcumin conjugated with hyaluronic acid HA	Wounds of mice treated with 20 ml of 210 mg/ml of hyaluronic (HA) or 20 ml of the 25 mM of curcumin or hyaluronic-curcumin (HA–cur)	Diabetic Swiss male albino mice Excision wounds	Curcumin topical effect enhanced wound healing compared to treatment with HA-free curcumin and HA alone	Sharma et al. 2018
19	Cinnamaldehyde	Male diabetic (BSK.Cg-m + / + Leprdb; db/db) and WT mice (C57BL/6 J), and male Kunming mice. Doses of intraperitoneal injection of cinnamaldehyde (25, 50, and 100 mg/kg)	Mice were injured with excisional skin wounds. Normal and diabetic mice were used in the study	Cinnamaldehyde-induced angiogenesis and led to an increased rate of wound repair	Yuan et al. 2018
20	Bromelain pineapple (Ananas comosus)	Bromelain was given intraperitoneally in doses of 25 mg/kg or 45 mg/kg	Full thickness incision and diabetic Male Wistar rats wound model	Bromelain significantly enhanced wound contraction and strength, reduced granulation tissue formation, and increased angiogenesis	Fathi et al. 2020
21	Bromelain pineapple (Ananas comosus)	Chitosan nanofibers loaded with bromelain were investigated in burn wound repair	Induced burn wounds in rats	The safety significantly improved, good impact on re-epithelialization, reduction of necrosis, and good wound closure were observed	Kalalinia et al. 2021
22	Luteolin	Intraperitoneal administration of luteolin 100 mg/kg body weight	Excision wounds diabetic male Wistar rat model	Wounded and diabetic rats experienced wound restoration via improving inflammatory and oxidative stress through the administration of luteolin	Chen et al. 2021
23	Luteolin	An ointment of luteolin of different concentrations (0.5% and 1% w/w) was applied topically on wounds	Excision and incision diabetic and nondiabetic male Wistar rate models	Luteolin ointments ameliorated wounds and enhanced skin tissue's healing process in both nondiabetic and diabetic wounds	Özay et al. 2018
24	Luteolin	Medical vaseline ointment of 10% luteolin	Skin wound of scald model males specific-pathogen-free Sprague Dawley rats	Inflammation of scalded rats was efficiently reduced with the promotion of proper wounds in luteolin treated group	Wang et al. 2022
25	Thymoquinone	0.5% w/w of thymoquinone nano-emulgel incorporated with Carbopol 940 (TMQ-NEG)	Excision wounds Wistar rats	The examined nano emulgel showed a faster and better wound-healing effect compared to the ordinary hydrogel form of thymoquinone	Algahtani et al. 2021
26	Thymoquinone Thymoquinone loaded chitosan-lecithin micelles	An investigation was done using 20 mg/mL of thymoquinone loaded to micelles formulation and with the 2% w/ w thymoquinone loaded to polymeric micelle-hydrogel	Excision wound model of old Balb/c mice	The hydrogel showed a remarkable wound-curing impact on the original thymoquinone and silver sulphadiazine	Negi et al. 2020
27	Thymoquinone	The polyvinyl pyrrolidone (PVP) matrix-type films containing 20% w/w of TQ were tested (hydrogel formulation)	Full-thickness excisional wound infection model in male mice (BALB/c)	TQ-containing films exhibited significant activity against *Staphylococcus aureus* infection	Haq et al. 2020
28	Gentiopicroside and Thymoquinone	Mats of co-blended polyvinyl pyrrolidine (PVP) and methyl ether Polyethylene glycol (m-PEG) were loaded with gentiopicroside and thymoquinone	White albino male rats were used	The polymeric mats are loaded with gentiopicroside and thymoquinone, so it could be considered suitable wound dressing	Almukainzi M. et al. 2022
29	Vicenin-2 (VCN-2)	VCN-2 in the form of hydrocolloid film	Wounds were inflicted in diabetic male adult Sprague Dawley rats	VCN-2 may have a wound-healing impact as wound treatment with VCN-2 hydrocolloid films could efficiently enhance wound repair in hyperglycemic cases	Tan et al. 2019
30	Kaempferol (KM)	The KM ointments 1% w/w were used	Diabetic excisional and nondiabetic incisional male Wistar rats' models	Kaempferol was an efficient wound-healing drug in treating both nondiabetic and diabetic wounds	Özay et al. 2019
31	Glycyrrhizin micelle as a genistein nanocarrier	Dipotassium glycyrrhizinate-based micelle ophthalmic solution encapsulating genistein (DG-Gen) 1:15	Diabetic corneal and nerve-wounded C57BL/6 J male mice	Application of the DG-Gen significantly prompted corneal re-epithelialization and nerve regeneration in wounded diabetic mice	Hou et al. 2021
32	Quinoline	A hydrogel loaded with Cu (II) Schiff base 8-hydroxy quinoline complex (CuSQ) solid lipid nanoparticles (SLN)	excision wound healing model in male Wistar albino rats	CuSQ would have a good impact as a drug for cutaneous wound curing through the control of growth factors and different cytokines	El-ezz et al. 2022
33	Micro-channeled alkylated chitosan sponge (MACS)	Liver perforation in male Wistar rats and Bama miniature male pigs was performed in this study	Pigs	The Micro-channeled alkylated chitosan sponge introduces higher pro-coagulant and hemostatic effects in lethal conditions of either normal or heparinized animal models. Generally, the MACS displayed promising clinical translational ability in managing fatal noncompressible hemorrhage and improving wound healing	Du et al. 2021
34	Green tea catechin (–)-Epigallocatechin-3-*O*-gallate (EGCG)	EGCG-grafted water-soluble silk fibroin hydrogels (SFEGCG). SFEGCG conjugate was co-crosslinked with tyramine-substituted SF (SF-T) via horseradish peroxidase (HRP)/H_2_O_2_ mediated enzymatic reaction to form SF-T/SF-EGCG hydrogels	Male Sprague Dawley rat model of full-thickness skin defect	SF-T70/SF-EGCG30 hydrogels exerted a remarkable wound-healing effect over SF-T hydrogels and a commercial DuoDERM® gel dressing	Lee et al. 2022

Botanical extracts have been extensively utilized in managing wounds in traditional medicine. Therefore, in vitro and in vivo studies have assessed different extracts for their wound-curing characteristics. Their phytochemical content is the purpose of their remedial features in wound repair. Other phytochemicals and plant-derived substances were investigated for their wound-healing activity as flavonols, flavanones, isoflavones, flavanols, flavonolignans, proanthocyanidins (Carvalho et al. 2021), β-glucans (Majtan and Jesenak 2018), bromelain (Fathi et al. 2020), curcumin (Akbik et al. 2014). It was disclosed that different botanicals and medicinal plants are widely used as a topical treatment for wound repairing, such as aloe vera, banana leaves (Sivamani et al. 2012), turmeric, *Centella asiatica*, *Rosmarinus officinalis, Calendula officinalis* (Artem Ataide et al. 2018).

Natural products such as plant extracts and other plant-derived products and their phytochemicals assist in managing inflammatory diseases, exert antimicrobial effects, and might aid skin tissue regeneration (Alherz et al. 2022; Attallah NG et al. 2022). They could remove oxidative stress and lower inflammation (Shah and Amini-Nik 2017). The wound-repairing ability of different plant extracts and their actives was confirmed in wound-curing animal models. Such plants improved collagen deposition, the proliferation of epithelial cells, and angiogenesis in diabetic and nondiabetic animal models (Binsuwaidan et al. 2022). Different types of plants are widely used in managing wounds and injuries from previous scientific research (Chingwaru et al. 2019).

The current review demonstrates and focuses on the latest findings in the last 5 years (2018–2022) regarding the in vivo studies of wound repairing effect of different plant extracts, the derived substances from plants, and pure natural substances as a new frontier in treating wounds.

## Methods of collecting data

Data collected in the frame of this work were generated by common research engines such as ScienceDirect, Web of Science, PubMed, SciFinder-n, and Scopus, using the references “natural products”, “wound healing” and refining with keywords “animal models”, “burns”, “biological”, “plants” “wound dressings” and “inflammation”. A total of 2194 research items were examined out of which 190 fall into the scope of the review, thus, constituting the baseline of the current survey.

### Botanicals and pure natural substances in the preclinical studies

The present review provided the research work, which included the preclinical studies (in vivo) of plant extracts and pure natural substances on wound healing in the last 5 years. The preclinical investigation by using animal models is important for acute and chronic wounds, in vitro studies could be used, but they do not assess the complexity of the wound healing process (Dunn et al. 2013; Zindle et al. 2021). Acute wounds occur through known sequential steps (Zindle et al. 2021). but chronic wounds exhibited impaired or delayed healing. The acute wound heals within 2–3 weeks, followed by the remodelling phase in normal healthy people. The normal healing sequence could be interrupted by other diseases such as diabetes, wound infection, foreign bodies, chronic inflammation, and ischemia. Microbial infection is the famous reason for wound-related morbidity (Said et al. 2009; Rajendran et al. 2018). This led to a physiological imbalance in the mechanism of healing. It might get stuck in one of the phases, and the wound then falls into the non-healing chronic type (FrykbergRobert 2015; Rajendran et al. 2018). It was reported that a wound is not healed in more than 6–8 weeks defined as a chronic/ non-healing wound (Rajendran et al. 2018). The universal goal of all studies about wound healing is to treat acute wounds perfectly in due time, so we avoid conversion into chronic ones and discover the appropriate therapy if the patient suffers from chronic wounds. Patients with chronic wounds suffer from pain, depression due to isolation from the community, and risk of amputation (Ivanková and Belovičová 2020).

### Wound healing potentials of various plant extracts

Different studies of the wound-repairing effect of various plant extracts revealed the diversity of actives responsible for this activity. It was suggested that D-pinitol and caffeic acid, the major constituents of *Boerhavia diffusa* leaf methanol extract, contributed to the wound-healing effect (Juneja et al. 2020). In another study, the fraction contained a high level of polyphenolic compounds, separated from leaves methanol extract of *Coccinia grandis* showed a remarkable wound repair effect. This effect was due to (rutin), quercetin-3-*O*-neohesperidin, nicotiflorin, kaempferol-3-*O*-glucorhamnoside, and astragalin as well as seco-iridoids of oleuropein and ligstroside (Al-Madhagy et al. 2019). HPLC metabolic profiling of the methanol extract of *Ephedra ciliata* recognized quercetin as a major compound. The antioxidant and antimicrobial activities of quercetin were related to the wound-closure effect of the extract (Yaseen et al. 2020). Biological guided study of *E. characias* subsp. *wulfenii* extracts (methanol, *n*-hexane, and ethyl acetate) of the aerial parts were tested. It was explored that the methanol extract displayed significant wound-repairing activity in circular excision and linear incision wound models, as well as anti-inflammatory effects. This study explored whether quercetin derivatives (quercitrin, hyperoside, and guaijaverin) were responsible for the wound-repairing effect (Özbilgin et al. 2018). Regarding *Jacaranda decurrens* Cham., metabolic profiling was done to find out ten compounds in the extract of flavonoidal and triterpenoidal nature. It was concluded that these compounds improved the healing of wounds in this study (Serra et al. 2020). Hydroethanolic extract of leaves of *Lafoensia pacari* A. St.-Hil. was evaluated in accelerating the contraction of wounds. The plant contained punicalagin, ellagic acid, punicalin, kaempferol, quercetin-3-*O*-xylopyranoside, and quercitrin, which could be related to re-epithelialization, improved cell proliferation, and enhanced remodeling phase of the wounds (Pereira et al. 2018). The mats composed of polyurethane loaded with *Nigella sativa* oil were studied to assess the in vivo wound-repairing effect (Aras et al. 2021). The essential oil of *Nigella sativa* seeds contains thymoquinone, which was reported to have wound-healing activity (Haq et al. 2020). Different studies were performed to obtain an effective wound healing process e. g. loaded thymoquinone chitosan- lecithin micelles which keep thymoquinone at the site of wounds with controlled release of the drug (Negi et al. 2020). Hydro-ethanol extract from *Vitis labrusca* leaves was found to advance the healing of wounds due to the total phenolic and flavonoid content (Santos et al. 2021). Aqueous ethanol extract of Leaves of *Curatella americana* Linn. exerted remarkable wound healing properties due to its active constituents. Leaves contain compounds known as wound-healing agents, mainly quercetin, kaempferol, glucosides, catechin, and epicatechin (Fujishima et al. 2020). A homogenous polysaccharide was separated from the rhizomes of *Curcuma zedoaria* and tested in the process of healing wounds in diabetic rats. It was added with platelet-rich plasma exosomes and loaded to a hydrogel sponge of chitosan and silk. It was found that the previous combination was effective and safe to speed the curing of wounds in the case of diabetes (Xu et al. 2018). Methanol extract of *Dodonaea viscosa* leaves caused accelerated epithelization of excision wounds and increased tensile strength of incision wounds of rats. HPTLC chromatogram showed 10 constituents of flavonoids, tannins, and saponins, including rutin and kaempferol, with reported healing effects (Nayeem et al. 2021).

### bio- and synthetic polymers of bioactive substances from natural products

Wound dressings can be created from a combination of bio- and synthetic polymers. Loading them with bioactive substances from natural products increased the good features of this combination. The combined bio- and synthetic polymers may have little or no anti-bacterial, anti-inflammatory, and antioxidant effects (Alven et al. 2020). Loading the bioactive natural product to either the combined polymers or to only one of them eliminates this problem. Bioactive materials such as curcumin (Lüer et al. 2012; Tejada et al. 2016), quercetin (Choudhary et al. 2020; Karuppannan et al. 2022), rutin (Zhou et al. 2021), bromelain (Kalalinia et al. 2021), thymoquinone, gentiopicroside (Almukainzi M. et al. 2022; Almukainzi May et al. 2022), hesperidin (Carvalho et al. 2021), and others were reported to enhance wound healing by adding them to bio- or synthetic polymers or both.

Different types of wound dressings have existed as traditional or passive, e.g., plasters and wool dressing which are not favorable nowadays because of the pain and possible re-skin damage. The interactive wound dressing of synthetic or bio-polymers could be represented as hydrogel, foams, sprays, films, and nanofibers, which introduced a moist environment for wound healing and facilitated water vapor transmission but with a limited anti-bacterial effect. Bioactive wound dressings could be represented by the previously mentioned types of interactive wound dressings, which may be composed of synthetic polymers of polyethylene glycol, polyvinyl pyrrolidone polyurethanes, poly-hydroxyethyl methacrylate, polyglycolic acid, polylactide, poly-ε-caprolactone, as well as biopolymers of pectin, chitosan, cellulose, dextran, and alginate, collagen, which are loaded with antibiotics or growth factors or vitamins, and/or bioactive natural products (Zahedi et al. 2010; Aderibigbe and Buyana 2018; Alven et al. 2020).

The merits of combining synthetic and bio-polymer with bioactive natural products in wound dressings for better wound healing were confirmed in many studies e.g., curcumin (Sharma et al. 2018), quercetin, and rutin (Zhou et al. 2021). Curcumin is the active substance of the roots of turmeric or *Curcuma longa.* It exerts strong antioxidant and anti-inflammatory, anti-bacterial effects but with low water solubility and oral bioavailability. Curcumin was loaded into bio- and synthetic polymers to overcome this problem (Alven et al. 2020). The combination between bio- and synthetic polymers could overcome the problem of poor mechanical support of bio-polymers (Aycan et al. 2019), besides overcoming the problem of lacking biocompatibility, biodegradability, and bad patient compliance of synthetic polymers (Mir et al. 2018). Effective wound dressing for skin burns represents a challenge to the healthcare system due to the probability of skin structure damage leading to an increased risk of infection. Quercetin and rutin are flavonoids with strong antioxidant, antimicrobial and anti-inflammatory effects but have limited water solubility. It was revealed that incorporating quercetin and rutin into polycaprolactone and chitosan oligosaccharides to form a new bioactive electrospun nanofiber membrane, exhibited superior efficacy among all nanofiber membranes for burn injuries (Zhou et al. 2021).

Regarding diabetic wounds, new scaffolds formed of polyethylene glycolylated graphene oxide collagen hybrid for nanoscale drug delivery of quercetin were tested. It was found that it provided a new scaffold with the advantages of being superior, stable, the controllable release of quercetin, biodegradable nanomaterial, and biocompatible, which permitted collagen formation and angiogenesis. Besides, the mesenchymal stem cells' proliferation and differentiation potential were promoted via adhesion to this scaffold. These new scaffolds could help in solving issues of deficient collagen hyperplasia and insufficient blood supply in the case of diabetic wounds (Chu et al. 2018).

## Conclusions and future direction

The current review clarifies that nature introduces medicinal plants with remarkable wound-healing effects. Scientific evidence obtained in the last 5 years has allowed us to expand our knowledge about herbal medicines on wound healing and the underlying molecular mechanisms. Plants, with their natural actives, have the ability to cure wounds and to be utilized in skin wound care. Mainly due to their anti-inflammatory, antimicrobial, and antioxidant activities (Pazyar et al. 2014).

Recent literature has proved that different natural substances, such as flavonoids, saponins, phenolic compounds, and polysaccharides, can operate at various phases of the process through diverse mechanisms and are primarily responsible for the activity of herbal remedies active in wound healing. Polyphenolic compounds have been confirmed therapeutical agents in wound healing by regulating and modulating inflammatory responses. Numerous phytochemicals in medicinal plants have been revealed to be important regulators of homeostasis, re-epithelialization, and regeneration by encouraging fibroblast proliferation and/or collagen formation. Scientific research confirmed the powerful impact of medicinal plants and their phytochemicals in wound management through multiple connected mechanisms (Maver et al. 2015; Artem Ataide et al. 2018).

The development of novel wound care techniques that integrate herbal healing agents with modern products and procedures is in line with current trends in wound healing. Nanostructures and nanoformulations have recently shown promise in overcoming the limitations of conventional medications. They control the release of medicines, lower the dosages needed for healing, and enhance the solubility and effectiveness of water-insoluble herbal components in healing wounds. The optimal dressing for wound treatment is made of nanofibers due to their well-controlled porosity and resemblance to skin tissue. The incorporation of natural materials into nanofibrous architectures for wound dressing has been studied. A biocompatible formulation made of natural herbal extracts would give the consumer a “green” option, and almost fewer side effects once put on the skin.

Based on these findings, it is recommended that many therapeutic approaches be employed concurrently in managing wounds, especially chronic wound injuries, to speed up the healing process and prevent complications. Moreover, various problems need to be resolved to improve the efficacy and utilization of natural substances in wound healing. Multidisciplinary efforts are required to confirm the products’ safety, look at their adverse effects, and do double-blind controlled clinical trials. Good production standards and regulatory regulations are equally essential to increase practitioners’ use of phytotherapy and encourage its incorporation into national health systems.

## Data Availability

The authors confirm that the data supporting this study are available within the article.
